# Ankle Kinematics and Neuromuscular Activity During the Stair‐to‐Ground Transition in Individuals With Acute Ankle Sprain: A Cross‐Sectional Study

**DOI:** 10.1002/jfa2.70221

**Published:** 2026-09-26

**Authors:** Haowen Sun, Qing Chen, Yanhua Xie, Zhenzhong Wang

**Affiliations:** ^1^ School of Physical Education Hanshan Normal University Chaozhou China; ^2^ School of Physical Education Hubei University of Science and Technology Xianning China

**Keywords:** acute ankle sprain, center of mass, intermuscular coherence, muscle activation, stair‐to‐ground transition

## Abstract

**Background:**

Evidence regarding the biomechanics of stair‐to‐ground transitions after acute ankle sprain (AAS) remains limited. This study examined ankle kinematics, center‐of‐mass (COM) displacement, muscle activation, and intermuscular coherence during this transition.

**Methods:**

Thirty individuals with AAS and 30 healthy controls completed the task while motion‐capture, force‐plate, and surface electromyography data were collected. Ankle kinematics were derived from motion capture, and COM displacement was obtained using a participant‐scaled OpenSim model. Trial‐level outcomes were averaged within each participant.

**Results:**

At initial contact, the AAS group showed an ankle position that was 1.622° more plantarflexed and 2.661° more internally rotated than that of the control group. Transverse‐plane COM displacement was 0.004 m lower in the AAS group (*p* = 0.011), whereas ankle range of motion did not differ between groups. Mean biceps femoris activation was lower, whereas mean medial gastrocnemius and tibialis anterior activation and peak tibialis anterior and soleus activation were higher in the AAS group (*p* < 0.05). In the AAS group, global efficiency, clustering coefficient, and network density were higher in the 0–25 Hz band than in the 25–50 Hz band (*p* < 0.05).

**Conclusions:**

AAS was associated with altered ankle posture at initial contact, a smaller transverse‐plane COM displacement amplitude, and differences in selected muscle‐activation outcomes during the stair‐to‐ground transition.

## 1 Introduction

Acute ankle sprain (AAS) is a common musculoskeletal injury. In the acute stage, pain, swelling, and functional difficulty may constrain loading. However, the clinical presentation and subsequent course are heterogeneous [1, 2, 3, 4]. AAS and chronic ankle instability (CAI) are distinct clinical states [5]. CAI is conceptualized within an updated framework involving interacting mechanical, sensory‐perceptual, and motor‐behavioral impairments following ankle sprain. These chronic features and corresponding CAI selection criteria should not be applied retrospectively to the present acute sample.

Stair descent requires foot placement, load acceptance, and control of the center of mass (COM) [6]. During the stair‐to‐ground transition, initial contact (IC) and early support are relevant because the lower limbs must accommodate descent while accepting body weight [7]. Evidence on stair descent after ankle injury has primarily concerned individuals with a history of ankle sprain or CAI rather than those with AAS [8, 9]. Related evidence in individuals with AAS has mainly been derived from landing tasks rather than stair descent [10]. Accordingly, the present knowledge gap is the concurrent characterization of ankle posture at IC, ankle excursion, COM displacement, and muscle behavior during the stair‐to‐ground transition in individuals with AAS.

Surface electromyography (sEMG) quantifies the amplitude and timing of individual‐muscle activity, whereas intermuscular coherence assesses frequency‐specific coupling between muscle signals and can describe shared oscillatory components across a muscle set [11, 12]. Network analysis summarizes the distribution of such pairwise coherence across multiple muscles and therefore provides complementary organizational information rather than a substitute for sEMG amplitude analysis [13]. The ankle was the primary kinematic focus because it was the injured joint. Muscles crossing the knee, hip, and ankle were recorded to describe activity distributed across the lower limb during load acceptance and COM regulation [14].

This cross‐sectional biomechanical study compared individuals with AAS and healthy controls during the stair‐to‐ground transition. We examined three‐dimensional ankle kinematics, COM displacement, muscle activation, and intermuscular coherence network characteristics. We hypothesized that the AAS group would show altered ankle posture at IC, lower transverse‐plane COM displacement, and altered activation of selected peri‐ankle muscles.

## 2 Methods

### 2.1 Participants

A sample‐size calculation was performed using G*Power version 3.1.9.7 for a two‐sided independent‐samples *t* test comparing two groups [15]. Assuming a large standardized mean difference of Cohen's *d* = 0.80, an α level of 0.05, and 80% power, 26 participants per group were required. Thirty participants per group were recruited to allow for potential data loss.

The study included 30 participants with unilateral AAS and 30 healthy controls. Mean age, height, and body mass were 22.70 ± 3.50 years, 174.8 ± 6.7 cm, and 69.8 ± 13.5 kg in the AAS group and 21.80 ± 4.25 years, 173.5 ± 5.8 cm, and 64.5 ± 12.8 kg in the control group, respectively. All participants were able to complete the stair‐descent task without assistance.

The inclusion criteria for the AAS group were unilateral AAS within the preceding 7 days, pain or perceived instability during daily activity or exercise, an American Orthopaedic Foot and Ankle Society (AOFAS) ankle‐hindfoot score below 80, a visual analog scale pain score no greater than 3 on the affected side, and the ability to descend stairs without assistance [16]. The 7‐day window was a study‐specific operational criterion used to restrict assessment to the early postinjury period rather than a validated cutoff separating acute from later recovery stages. The AOFAS ankle‐hindfoot scale includes assessments of pain, function, and alignment [17]. A cutoff below 80 was used as a study‐specific indicator of ankle‐related functional limitation rather than as a validated diagnostic or injury‐severity threshold. The pain threshold was not used to diagnose AAS or classify injury severity; together with the unaided stair‐descent requirement, it was used to identify participants who could tolerate the loading task without severe pain. The AOFAS and pain thresholds were used solely for eligibility and were not treated as study outcomes or used to define clinical subgroups.

AAS diagnosis was based on injury history and clinical assessment. Mechanical joint laxity was not quantified. Sprain grade, specific ligament involvement, swelling severity, previous sprain history, and exact injury‐to‐test day within the 7‐day eligibility window were not available in the retained analysis dataset. The AOFAS ankle‐hindfoot and pain scores were used only to confirm eligibility and were not retained in the analysis dataset as study outcomes. Accordingly, the AAS participants were analyzed as a single clinically heterogeneous cohort, and relationships between these clinical characteristics and the biomechanical or neuromuscular outcomes could not be evaluated. Participants were excluded if they had a suspected fracture, complete ligament rupture, previous ankle surgery or fracture, another lower‐limb injury affecting movement, a neurological or neuromuscular disorder, structured lower‐limb rehabilitation within the preceding 6 months, or an inability to complete the stair‐descent task. Control participants were required to have no recent acute lower‐limb injury, chronic ankle instability, neurological or neuromuscular disorder, or pain during stair descent.

All participants provided written informed consent before testing. The study was approved by the Ethics Committee of Hanshan Normal University, approval number 2026010809, and was conducted in accordance with the Declaration of Helsinki.

### 2.2 Experimental Setup and Procedures

All experiments were conducted in the Biomechanics Laboratory. Three‐dimensional kinematic data were captured using an eight‐camera Vicon motion‐capture system (version 2.15.0; Oxford, UK) at 200 Hz. Ground‐reaction‐force data were recorded synchronously using two embedded Kistler force plates at 1000 Hz, and sEMG signals were collected using a wireless Delsys sEMG system at 2000 Hz. All data streams were synchronized through a single workstation. The stair‐descent landing protocol was developed for the present laboratory study and was not reproduced from a previously published standardized protocol. It was designed to examine the final stair‐to‐level‐ground transition, during which the tested limb accepted body weight on the embedded force plate. The task was performed on a custom‐built staircase without handrails, with a riser height of 0.20 m and a tread depth of 0.26 m. The analyzed maneuver was the forward transition from the final stair tread to the level‐ground landing area. A designated force plate was embedded within the level‐ground landing area following the final tread.

The affected limb was tested in the AAS group. In the control group, the tested limb was assigned to approximate the left–right distribution of the affected limbs in the AAS group, thereby reducing systematic side imbalance between the groups. Limb dominance was not assessed and was not used to select the tested limb. After ultrasound‐assisted identification of the muscle bellies and standardized skin preparation, wireless sEMG sensors were placed over the rectus femoris (RF), vastus medialis (VM), vastus lateralis (VL), biceps femoris (BF), semimembranosus (SM), medial gastrocnemius (MG), lateral gastrocnemius (LG), tibialis anterior (TA), soleus (SOL), and gluteus maximus (GMax). Sensor placement followed SENIAM principles [18].

Reflective‐marker trajectories were acquired for the calculation of three‐dimensional ankle angles and participant‐specific scaling of the generic OpenSim Gait2392 model [19]. The motion‐capture and OpenSim workflows were used for distinct outcomes: ankle angles were calculated from the processed motion‐capture data, whereas the participant‐scaled Gait2392 model was used to estimate whole‐body COM. Each participant completed a static calibration trial, which was used to scale the model before inverse kinematics was applied to the dynamic trials. Participants completed five familiarization trials followed by 10 min of seated rest. During formal testing, participants descended the staircase without external assistance at a self‐selected comfortable speed. Movement speed was not monitored or standardized. Footwear and the use of external ankle support were neither standardized nor systematically recorded. During the final transition from the staircase to level ground, participants were instructed to place the tested foot fully on the designated force plate within the landing area. Only the final stair‐to‐level‐ground transition and the subsequent landing support phase of the tested limb were included in the analysis [20]. The preceding stair‐descent steps and the pre‐contact phase were not analyzed.

### 2.3 Data Collection

Before formal testing, three maximum voluntary isometric contraction (MVIC) trials were recorded for each target muscle. Each trial lasted 3–5 s, with a 1‐min rest interval between trials. For each muscle and participant, the largest MVIC‐derived reference value across the three trials was used for amplitude normalization. MVIC‐based normalization expresses task‐related sEMG amplitude relative to an individual reference contraction [21].

A trial was considered valid if the tested foot fully contacted the designated force plate, the movement was completed naturally without obvious adjustment or pause, and key reflective markers remained visible throughout the landing support phase. Trials failing these validity criteria were not retained. At least five valid trials were retained for each participant, and variables extracted from valid trials were averaged for statistical analysis.

### 2.4 Data Processing

Raw marker‐trajectory data were processed in Vicon Nexus version 2.15.0, including marker identification and gap filling. The landing support phase was defined from IC to toe‐off of the tested foot. IC and toe‐off were identified when the vertical ground‐reaction force exceeded and subsequently fell below 20 N, respectively. Processed marker trajectories and the corresponding static calibration trial were then imported into OpenSim version 4.5. The generic Gait2392 model was scaled to each participant using the static trial, after which OpenSim inverse kinematics estimated the model coordinates at each frame by minimizing the weighted least‐squares error between the experimental and model‐marker positions [22]. The resulting model motion was used to estimate the whole‐body COM position. Ground‐reaction‐force data were used only to identify the analysis phase and were not used for model scaling, inverse kinematics, or COM calculation.

Kinematic and ground‐reaction‐force data were filtered using fourth‐order zero‐lag Butterworth low‐pass filters at 6 and 40 Hz, respectively. Three‐dimensional ankle angles were calculated separately from the processed motion‐capture data and were not based on the constrained ankle and subtalar coordinates of the Gait2392 model. Positive sagittal‐, frontal‐, and transverse‐plane values represented dorsiflexion, eversion, and external rotation, respectively, whereas negative values represented plantarflexion, inversion, and internal rotation [23]. Ankle angle at the IC and ankle range of motion were extracted during the landing support phase.

Amplitude analysis of sEMG signals was performed in MATLAB. Signals were segmented to the landing support phase, filtered using a fourth‐order Butterworth band‐pass filter at 20–450 Hz, full‐wave rectified, and converted to linear envelopes using a 6‐Hz low‐pass filter. For each muscle, the envelope was normalized to that participant's recorded MVIC reference and expressed as a percentage of MVIC. Mean activation was the time average of the normalized envelope over the landing support phase, and peak activation was its maximum. Mean and peak sEMG amplitudes were calculated separately for each muscle. No co‐activation or co‐contraction index was calculated. Percentage‐of‐MVIC values represent sEMG amplitude relative to the selected reference contraction and should not be interpreted as direct measurements of absolute muscle force or neural drive [24].

Whole‐body COM position was obtained from the scaled OpenSim model motion and therefore reflected the model segment mass properties and inverse‐kinematics‐derived pose. Displacement amplitudes in the sagittal, frontal, and transverse planes were extracted during the landing support phase.

Intermuscular coherence analysis was performed separately from sEMG amplitude analysis. Coherence was estimated from unrectified sEMG without the 6‐Hz linear‐envelope step, avoiding the nonlinear spectral distortion introduced by rectification. These signals were filtered using a fourth‐order Butterworth band‐pass filter at 5–450 Hz and segmented to the landing support phase. Coherence was calculated between each pair of the 10 target muscles (RF, VM, VL, BF, SM, MG, LG, TA, SOL and GMax), producing 45 muscle pairs. The network analysis was restricted to the 10 muscles included in the predefined acquisition protocol. Because the peroneal muscles and gluteus medius were not recorded, the network was intended to characterize coupling within the recorded muscle set rather than provide a complete representation of lateral ankle stabilization or proximal frontal‐plane control.

For each muscle pair and valid trial, magnitude‐squared coherence was estimated using Welch's method, with a 500‐ms Hann window, 50% overlap, and a 1000‐point fast Fourier transform. At the 2000‐Hz sampling rate, each window contained 1000 samples and provided a frequency‐bin spacing of 2 Hz. Coherence was calculated over the 0–50 Hz frequency range:

(1) $$C_{\text{xy}}\left(f\right)=\frac{|P_{\text{xy}}\left(f\right){|}^{2}}{P_{\text{xx}}\left(f\right)P_{\text{yy}}\left(f\right)}$$

where $C_{\text{xy}}\left(f\right)$ denotes the magnitude‐squared coherence between muscle signals x and y at frequency f. $P_{\text{xx}}\left(f\right)$ and $P_{\text{yy}}\left(f\right)$ denote the autospectral densities of signals x and y, respectively, and $P_{\text{xy}}\left(f\right)$ denotes their cross‐spectral density.

Following the methodological framework of Liang et al. [25], the coherence spectrum was summarized over the operational 0–25 Hz and 25–50 Hz frequency ranges. These broad ranges were selected to distinguish lower‐frequency common modulation, including beta‐range coupling, from coupling extending into higher beta and low‐gamma frequencies. They were treated as operational summary bands rather than frequency components estimated using nonnegative matrix factorization or as markers of distinct physiological generators. Because the sEMG signals were high‐pass filtered at 5 Hz, spectral content below 5 Hz in the nominal 0–25 Hz band was attenuated; therefore, the physiologically interpretable content of the lower band was effectively approximately 5–25 Hz. Neither frequency range was attributed to a unique neural pathway.

For each valid trial and frequency band, mean coherence was calculated for each of the 45 muscle pairs and arranged into a symmetric 10 × 10 weighted adjacency matrix. The 10 recorded muscles were represented as network nodes, pairwise coherence values were represented as edge weights, and diagonal elements were set to zero. Trial‐level coherence matrices and network outcomes were calculated separately and then averaged across valid trials with equal weight to obtain one participant‐level value for each frequency band. Individual muscle‐pair edges were not subjected to inferential significance testing and were therefore interpreted descriptively.

For network analysis, each muscle was represented as a node, and the mean coherence between the two muscles was used as the corresponding edge weight. Global efficiency was calculated from the weighted adjacency matrix. A fixed relative threshold equal to 30% of the maximum edge weight within each trial‐level matrix was used to construct the binary networks, following previous lower‐limb muscle‐network research. A relative threshold was selected to retain the strongest connections in relation to the weight distribution of each matrix rather than applying the same absolute coherence value across participants and trials. Edges with weights equal to or greater than the threshold were retained, whereas weaker edges were set to zero. The same rule was applied to all participants, trials, and frequency bands without data‐driven optimization. The 30% threshold was not assumed to represent a physiologically unique or empirically optimal cutoff. The resulting binary matrix was used to calculate the clustering coefficient and network density and to visualize network topology. Global efficiency was calculated as follows:

(2) $$E_{\text{glob}}=\frac{1}{N\left(N-1\right)}\sum_{i\neq j}\frac{1}{d_{\text{ij}}}$$

where N is the number of nodes, with N=10 in the present study, and $d_{\text{ij}}$ is the shortest weighted path length between nodes i and j. The mean clustering coefficient of the thresholded binary network was calculated as follows:

(3) $$C=\frac{1}{N}\sum_{i=1}^{N}\frac{2t_{i}}{k_{i}\left(k_{i}-1\right)}$$

where $t_{i}$ is the number of triangular connections around node i, and $k_{i}$ is the degree of node i in the thresholded binary network. The clustering coefficient of a node was defined as zero when $k_{i}<2$. Network density was calculated from the thresholded binary matrix as follows:

(4) $$D=\frac{2E}{N\left(N-1\right)}$$

where E is the number of retained undirected edges after application of the 30% threshold, and N is the number of nodes. With 10 muscles, the maximum possible number of undirected edges was 45.

**FIGURE 1:**
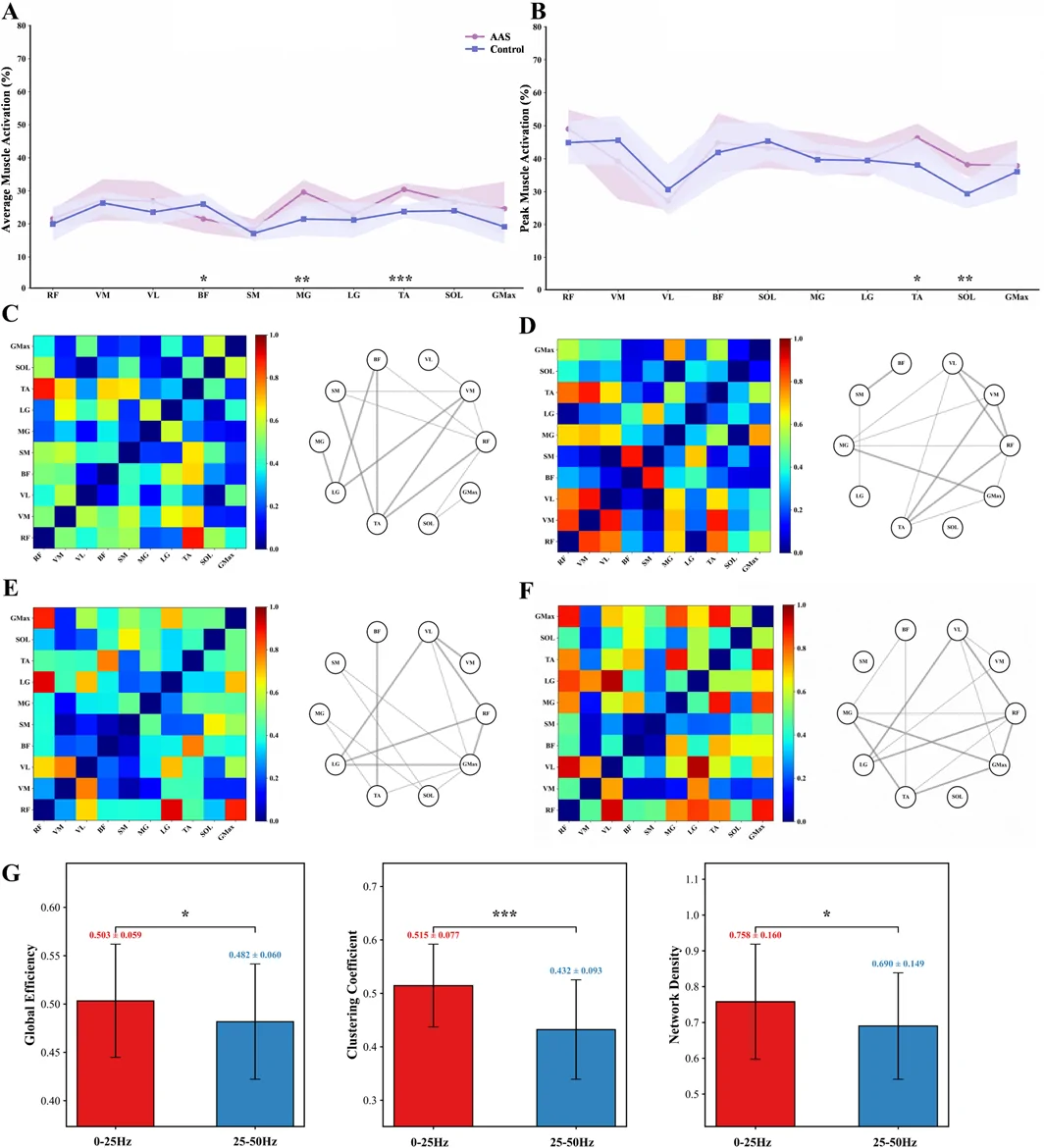
Muscle activation and intermuscular coherence during the stair‐to‐ground transition. (A, B) Mean and peak muscle activation, respectively (mean ± SD; %MVIC). (C, D) Coherence matrices and thresholded networks for the AAS group in the 0–25 Hz and 25–50 Hz bands, respectively. (E, F) Corresponding coherence matrices and thresholded networks for the control group. (G) Network indices within the AAS group (mean ± SD). Symbols indicate between‐group differences in A and B and within‐AAS frequency‐band differences in G: *p < 0.05, **p < 0.01, and ***p < 0.001.

For the group‐level coherence matrices shown in Figure 1, participant‐level matrices were averaged within each group. The same 30% relative‐threshold rule was then applied to each group‐average matrix to generate the corresponding network topology map.

### 2.5 Statistical Analysis

Statistical analyses were performed using SPSS version 26.0 (IBM Corp., Armonk, NY, USA) and MATLAB R2022a (MathWorks Inc., Natick, MA, USA). For each participant, trial‐level outcomes were averaged across all valid trials before statistical analysis. Normality was assessed using the Shapiro–Wilk test. Between‐group comparisons were performed using independent‐samples t tests for normally distributed variables and Mann–Whitney U tests for non‐normally distributed variables. The analyzed outcomes included ankle angle at initial contact, ankle range of motion, three‐dimensional center‐of‐mass displacement, mean muscle activation, and peak muscle activation.

Network indices included global efficiency, clustering coefficient, and network density. Within the AAS group, the 0–25 Hz and 25–50 Hz frequency bands were compared using paired‐samples t tests for normally distributed variables or Wilcoxon signed‐rank tests for non‐normally distributed variables. No multiplicity adjustment was applied across the biomechanical, sEMG, and network outcomes. Accordingly, all *p* values are nominal and unadjusted, and *p* < 0.05 was used as the reporting threshold.

## 3 Results

### 3.1 Ankle Kinematics and COM Displacement

Between‐group comparisons showed that the AAS group had a more plantarflexed ankle position at IC than the control group (−25.436° vs. −23.814°, mean difference −1.622°, *p* = 0.015). The AAS group also had a more internally rotated ankle position at IC (−4.002° vs. −1.341°, mean difference −2.661°, *p* < 0.001) (Table 1).

**TABLE 1:** Group means and between‐group comparisons of ankle kinematics and COM displacement.

Outcome	AAS group mean	Control group mean	Mean difference	*p* value
IC sagittal angle (°)	−25.436	−23.814	−1.622	0.015
IC frontal angle (°)	−1.108	−2.889	1.781	> 0.05
IC transverse angle (°)	−4.002	−1.341	−2.661	< 0.001
Sagittal ROM (°)	41.146	43.599	−2.453	> 0.05
Frontal ROM (°)	13.100	11.334	1.766	> 0.05
Transverse ROM (°)	6.454	5.493	0.961	> 0.05
Sagittal COM displacement (m)	0.156	0.159	−0.003	> 0.05
Frontal COM displacement (m)	0.113	0.085	0.028	> 0.05
Transverse COM displacement (m)	0.021	0.025	−0.004	0.011

*Note:* Values are group means. Mean differences were calculated as AAS minus control and are expressed in the original measurement units. All *p* values are nominal and unadjusted.

Abbreviations: AAS, acute ankle sprain; COM, center of mass; IC, initial contact; ROM, range of motion.

Transverse‐plane COM displacement was smaller in the AAS group than in the control group (0.021 vs. 0.025 m, mean difference −0.004 m, *p* = 0.011). No between‐group differences were observed in sagittal‐, frontal‐, or transverse‐plane ankle ROM. Group means, mean differences, and *p* values are presented in Table 1.

### 3.2 Muscle Activation and Intermuscular Coherence

Between‐group comparisons indicated differences in selected muscle‐activation outcomes during the landing support phase (Figure 1). Mean BF activation was 4.5% points of MVIC lower in the AAS group than in the control group (*p* = 0.041). Mean MG and TA activation were 8.0 and 6.6% points of MVIC higher in the AAS group, respectively (*p* = 0.004 and *p* < 0.001). Peak TA and SOL activation were also higher in the AAS group by 8.2 and 8.8% points of MVIC, respectively (*p* = 0.034 and *p* = 0.001).

Coherence matrices for both groups in the 0–25 Hz and 25–50 Hz frequency bands are presented descriptively in Figure 1. Individual muscle‐pair edges were not subjected to inferential significance testing. Within the AAS group, global efficiency was higher in the 0–25 Hz band than in the 25–50 Hz band (0.503 ± 0.059 vs. 0.482 ± 0.060, mean difference 0.021, *p* = 0.049). The clustering coefficient was also higher in the 0–25 Hz band (0.515 ± 0.077 vs. 0.432 ± 0.093, mean difference 0.083, *p* < 0.001), as was the network density (0.758 ± 0.160 vs. 0.690 ± 0.149, mean difference 0.068, *p* = 0.017). Figure 1 presents group means and corresponding SDs for the muscle‐activation outcomes and the within‐AAS network indices.

## 4 Discussion

This study examined ankle kinematics, COM displacement, muscle activation, and intermuscular coherence during the stair‐to‐ground transition in individuals with AAS. The AAS group showed more plantarflexed and internally rotated ankle positions at IC, lower transverse‐plane COM displacement, and altered activation of selected lower‐limb muscles. Ankle ROM did not differ between the groups. This pattern localizes the observed kinematic difference to IC rather than demonstrating reduced ankle excursion throughout the landing support phase.

The more plantarflexed and internally rotated IC posture may reflect a pain‐related protective or compensatory pre‐contact adjustment for load acceptance, as pain can produce task‐specific changes in motor behavior [26]. Because ankle ROM during the landing support phase did not differ between groups, this finding is more consistent with altered positioning at contact than with a demonstrated restriction of ankle motion. However, a more plantarflexed position should not be equated with greater stability. Plantarflexed and internally rotated or inverted ankle configurations have been associated with lateral ankle‐sprain mechanisms and may place the lateral ligament complex in a mechanically vulnerable position, depending on the loading context [27, 28]. The present data therefore identify an altered contact strategy but do not establish its cause or whether it is protective or detrimental. Because the analysis began at IC, it did not capture anticipatory muscle activity or limb positioning before contact.

Reduced transverse‐plane COM displacement may be compatible with a more constrained whole‐body movement pattern, but COM displacement alone is insufficient to establish improved dynamic stability and may also reflect altered movement fluidity or task execution [29]. Participants descended at a self‐selected comfortable speed. Stair‐negotiation speed can influence lower‐limb joint kinematics and muscle activation [30], and locomotor speed can also substantially affect COM displacement. Because movement speed was neither monitored nor standardized, between‐group differences in ankle kinematics, COM behavior, and muscle activation may partly reflect speed variation. The observed differences therefore cannot be attributed to AAS alone.

The muscle‐activation results showed higher mean MG and TA activation, higher peak TA and SOL activation, and lower mean BF activation in the AAS group. These differences may reflect altered muscle recruitment during load acceptance, but their functional meaning cannot be determined from sEMG amplitude alone [31]. Ankle stiffness was not measured, and muscle co‐activation or co‐contraction was not quantified. Therefore, the observed activation differences should not be interpreted as direct evidence of increased joint stiffness or greater muscular stabilization. Percentage‐of‐MVIC values represent sEMG amplitude relative to each participant's selected reference contraction. Because normalized values depend on the normalization task, testing posture, and reference procedure, they should not be interpreted as absolute neural drive; direct numerical comparisons across muscles also require caution because each muscle is scaled to a separate reference contraction. Uzlasir et al. compared individuals with CAI and healthy controls during jump landings at different inversion angles and reported impaired landing‐related proprioception but no overall between‐group differences in TA or MG activation [32].

The intermuscular coherence results characterize frequency‐specific coupling within the AAS group rather than between‐group differences in network organization. EMG–EMG coherence reflects frequency‐specific linear coupling between muscle signals and may provide an indirect measure of shared oscillatory input to the corresponding motor‐neuron populations [33]. However, coherence is non‐directional and cannot identify a unique anatomical or neural source [34]. The coherence matrices and thresholded networks therefore summarize coupling within the recorded muscle set; individual muscle‐pair edges were not subjected to inferential significance testing [35].

Taken together, the findings suggest that the stair‐to‐ground transition captures concurrent ankle, whole‐body, and muscle‐activation features during early AAS. The task may therefore provide a useful experimental paradigm for studying postinjury movement behavior, although it has not been validated as a clinical or prognostic assessment. Its use should be limited to individuals who can safely bear weight and complete stair descent with tolerable symptoms, consistent with the eligibility profile of the present sample [36].

This study has several limitations. First, its cross‐sectional design precludes causal inference, and the physically active university sample may limit generalizability. Sex was not included as an analytical factor, and the study was neither designed nor powered to evaluate sex‐specific effects. The AAS cohort was analyzed without stratification by sprain grade, specific ligament involvement, swelling severity, previous sprain history, or exact injury‐to‐test day within the 7‐day eligibility window. The findings therefore cannot be attributed to a particular injury severity, ligament‐injury pattern, or postinjury time point. Limb dominance was not assessed and may have influenced the observed between‐group differences. Stair descent was performed at a self‐selected speed without speed monitoring or standardization. Consequently, the potential effects of movement speed, footwear, and external support on ankle kinematics, COM displacement, and muscle activation could not be separated from the observed group differences.

Second, the analysis was restricted to the landing support phase and did not capture anticipatory neuromuscular activity or limb positioning before IC. Kinematic analysis was restricted to the ankle; consequently, the study cannot determine whether the observed ankle posture was accompanied by compensatory changes in knee or hip motion. The ankle‐angle and COM outcomes were derived using a laboratory‐specific motion‐capture and musculoskeletal‐modeling workflow and remain dependent on the implemented marker configuration, segment definitions, model‐scaling procedure, and inverse‐kinematics settings. The peroneal muscles and gluteus medius were not recorded, limiting inference regarding lateral ankle and proximal frontal‐plane control. Individual coherence edges were not subjected to inferential significance testing. Binary network topology, clustering coefficient, and network density depended on the selected 30% relative threshold. Because alternative thresholds and weighted‐network sensitivity analyses were not evaluated, the robustness of these outcomes across network‐construction choices is unknown. The binary‐network findings should therefore be interpreted as threshold‐dependent and exploratory. The available documentation of the reflective‐marker protocol may limit full reproducibility of anatomical landmark placement. Network indices were compared only between frequency bands within the AAS group; no between‐group inferential comparison of network indices was performed. Finally, testing multiple outcomes without multiplicity adjustment increased the risk of Type I error, particularly for findings close to *p* = 0.05.

## 5 Conclusion

This study expands the limited evidence on stair negotiation in acute ankle sprain through an integrated assessment of ankle mechanics, whole‐body movement, muscle activation, and intermuscular coherence during the stair‐to‐ground transition. The findings show that early postinjury movement behavior can be characterized across joint, whole‐body, and muscle‐activation domains, while coherence analysis provides complementary information on frequency‐specific coupling within the AAS group. This multimodal profile provides a basis for longitudinal studies examining recovery and clinical relevance.

## Author Contributions

**Haowen Sun:** conceptualization, investigation, data curation, writing – original draft, visualization. **Qing Chen:** methodology, data curation, writing – review and editing. **Yanhua Xie:** investigation, validation, data curation. **Zhenzhong Wang:** conceptualization, project administration, supervision, writing – review and editing.

## Funding

The authors have nothing to report.

## Ethics Statement

This study was approved by the Ethics Committee of Hanshan Normal University, with the ethics approval number 2026010809.

## Consent

Written informed consent was obtained from all participants before testing.

## Conflicts of Interest

The authors declare no conflicts of interest.

## Data Availability

The data that support the findings of this study are available from the corresponding author upon reasonable request.

## References

[1] C. Doherty , E. Delahunt , B. Caulfield , J. Hertel , J. Ryan , and C. Bleakley , “The Incidence and Prevalence of Ankle Sprain Injury: A Systematic Review and Meta‐Analysis of Prospective Epidemiological Studies,” Sports Medicine 44, no. 1 (2014): 123–140, 10.1007/s40279-013-0102-5. 24105612

[2] G. Vuurberg , A. Hoorntje , L. M. Wink , et al., “Diagnosis, Treatment and Prevention of Ankle Sprains: Update of an Evidence‐Based Clinical Guideline,” British Journal of Sports Medicine 52, no. 15 (2018): 956–970, 10.1136/bjsports-2017-098106. 29514819

[3] P. A. Gribble , C. M. Bleakley , B. M. Caulfield , et al., “Evidence Review for the 2016 International Ankle Consortium Consensus Statement on the Prevalence, Impact and long‐term Consequences of Lateral Ankle Sprains,” British Journal of Sports Medicine 50, no. 24 (2016): 1496–1505, 10.1136/bjsports-2016-096189. 27259753

[4] E. Delahunt , C. M. Bleakley , D. S. Bossard , et al., “Clinical Assessment of Acute Lateral Ankle Sprain Injuries (ROAST): 2019 Consensus Statement and Recommendations of the International Ankle Consortium,” British Journal of Sports Medicine 52, no. 20 (2018): 1304–1310, 10.1136/bjsports-2017-098885. 29886432

[5] J. Hertel and R. O. Corbett , “An Updated Model of Chronic Ankle Instability,” Journal of Athletic Training 54, no. 6 (2019): 572–588, 10.4085/1062-6050-344-18. PMC6602403 31162943

[6] A. Protopapadaki , W. I. Drechsler , M. C. Cramp , F. J. Coutts , and O. M. Scott , “Hip, Knee, Ankle Kinematics and Kinetics During Stair Ascent and Descent in Healthy Young Individuals,” Clinical biomechanics 22, no. 2 (2007): 203–210, 10.1016/j.clinbiomech.2006.09.010. 17126461

[7] T. Cluff and D. G. E. Robertson , “Kinetic Analysis of Stair Descent: Part 1. Forwards Step‐Over‐Step Descent,” Gait & Posture 33, no. 3 (2011): 423–428, 10.1016/j.gaitpost.2010.12.016. 21292489

[8] C. Doherty , C. Bleakley , J. Hertel , et al., “Lower Extremity Coordination and Symmetry Patterns During a Drop Vertical Jump Task Following Acute Ankle Sprain,” Human Movement Science 38 (2014): 34–46, 10.1016/j.humov.2014.08.002. 25240177

[9] B. Xuan , D. Sun , Y. Song , et al., “Lower‐Limb Biomechanical Alterations Across Movement Tasks in Individuals With Chronic Ankle Instability: An Umbrella Review of Systematic Reviews and meta‐analyses,” Mechanobiology in Medicine 4, no. 3 (2026): 100206, 10.1016/j.mbm.2026.100206. PMC13485636 42620678

[10] C. Doherty , C. Bleakley , J. Hertel , B. Caulfield , J. Ryan , and E. Delahunt , “Single‐Leg Drop Landing Motor Control Strategies Following Acute Ankle Sprain Injury,” Scandinavian Journal of Medicine & Science in Sports 25, no. 4 (2015): 525–533, 10.1111/sms.12282. 24975875

[11] L. Labanca , M. Mosca , M. Ghislieri , V. Agostini , M. Knaflitz , and M. G. Benedetti , “Muscle Activations During Functional Tasks in Individuals With Chronic Ankle Instability: A Systematic Review of Electromyographical Studies,” Gait & Posture 90 (2021): 340–373, 10.1016/j.gaitpost.2021.09.182. 34564008

[12] D. Farina , R. Merletti , and R. M. Enoka , “The Extraction of Neural Strategies From the Surface EMG: An Update,” Journal of Applied Physiology 117, no. 11 (2014): 1215–1230, 10.1152/japplphysiol.00162.2014. PMC4254845 25277737

[13] J. N. Kerkman , A. Daffertshofer , L. L. Gollo , M. Breakspear , and T. W. Boonstra , “Network Structure of the Human Musculoskeletal System Shapes Neural Interactions on Multiple Time Scales,” Science Advances 4, no. 6 (2018): eaat0497, 10.1126/sciadv.aat0497. PMC6021138 29963631

[14] B. Xuan , D. Sun , F. Li , et al., “The Impact of Cognitive Load on Lower Limb Biomechanics During the Take‐off Phase Before Service in Tennis Players of Different Levels,” Journal of Sports Sciences 44, no. 7 (2026): 896–906, 10.1080/02640414.2026.2619335. 41568817

[15] H. Kang , “Sample Size Determination and Power Analysis Using the G*Power Software,” Journal of Education Evaluation Health Prof 18 (2021): 17, 10.3352/jeehp.2021.18.17. PMC8441096 34325496

[16] D. A. Delgado , B. S. Lambert , N. Boutris , et al., “Validation of Digital Visual Analog Scale Pain Scoring With a Traditional Paper‐Based Visual Analog Scale in Adults,” Journal of the American Academy of Orthopaedic Surgeons 2, no. 3 (2018): e088, 10.5435/JAAOSGlobal-D-17-00088. PMC6132313 30211382

[17] A. S. de Boer , D. E. Meuffels , C. H. van der Vlies , et al., “Validation of the American Orthopaedic Foot and Ankle Society Ankle‐Hindfoot Scale Dutch Language Version in Patients With Hindfoot Fractures,” BMJ Open 7, no. 11 (2017): e018314, 10.1136/bmjopen-2017-018314. PMC5695419 29138208

[18] H. J. Hermens , B. Freriks , C. Disselhorst‐Klug , and G. Rau , “Development of Recommendations for SEMG Sensors and Sensor Placement Procedures,” Journal of Electromyography and Kinesiology 10, no. 5 (2000): 361–374, 10.1016/S1050-6411(00)00027-4. 11018445

[19] S. L. Delp , F. C. Anderson , A. S. Arnold , et al., “Opensim: Open‐Source Software to Create and Analyze Dynamic Simulations of Movement,” IEEE Transactions on Biomedical Engineering 54, no. 11 (2007): 1940–1950, 10.1109/TBME.2007.901024. 18018689

[20] J. L. Hicks , T. K. Uchida , A. Seth , A. Rajagopal , and S. L. Delp , “Is my Model Good Enough? Best Practices for Verification and Validation of Musculoskeletal Models and Simulations of Movement,” Journal of Biomechanical Engineering 137, no. 2 (2015): 020905, 10.1115/1.4029304. PMC4321112 25474098

[21] M. Besomi , P. W. Hodges , E. A. Clancy , et al., “Consensus for Experimental Design in Electromyography (CEDE) Project: Amplitude Normalization Matrix,” Journal of Electromyography and Kinesiology 53 (2020): 102438, 10.1016/j.jelekin.2020.102438. 32569878

[22] C. L. Dembia , N. A. Bianco , A. Falisse , J. L. Hicks , and S. L. Delp , “OpenSim Moco: Musculoskeletal Optimal Control,” PLoS Computational Biology 16, no. 12 (2020): e1008493, 10.1371/journal.pcbi.1008493. PMC7793308 33370252

[23] G. Wu , S. Siegler , P. Allard , et al., “ISB Recommendation on Definitions of Joint Coordinate System of Various Joints for the Reporting of Human Joint motion—Part I: Ankle, Hip, and Spine,” Journal of Biomechanics 35, no. 4 (2002): 543–548, 10.1016/S0021-9290(01)00222-6. 11934426

[24] B. Xuan , Y. Song , D. Sun , et al., “Optimizing Landing Mechanics to Modulate Patellar Tendon Loading: An Individualized Moment Arm Analysis,” iScience 29, no. 5 (2026): 115541, 10.1016/j.isci.2026.115541. PMC13099515 42028009

[25] T. Liang , H. Miao , H. Wang , X. Liu , and X. Liu , “Surface Electromyography‐Based Analysis of the Lower Limb Muscle Network and Muscle Synergies at Various Gait Speeds,” IEEE Transactions on Neural Systems and Rehabilitation Engineering 31 (2023): 1230–1237, 10.1109/TNSRE.2023.3242911. 37022413

[26] S. L. Merkle , K. A. Sluka , and L. A. Frey‐Law , “The Interaction Between Pain and Movement,” Journal of Hand Therapy 33, no. 1 (2020): 60–66, 10.1016/j.jht.2018.05.001. PMC6335190 30025839

[27] J. Hertel , “Functional Anatomy, Pathomechanics, and Pathophysiology of Lateral Ankle Instability,” Journal of Athletic Training 37, no. 4 (2002): 364–375. PMID: 12937557; PMCID: PMC164367. PMC164367 12937557

[28] D. T. P. Fong , K. M. Mok , I. M. Thompson , Y. Wang , W. Shan , and M. A. King , “A Lateral Ankle Sprain During a Lateral Backward Step in Badminton: A Case Report of a Televised Injury Incident,” Journal of Sport and Health Science 12, no. 1 (2023): 139–144, 10.1016/j.jshs.2021.03.007. PMC9923400 33744478

[29] S. M. Bruijn , O. G. Meijer , P. J. Beek , and J. H. van Dieën , “Assessing the Stability of Human Locomotion: A Review of Current Measures,” Journal of the Royal Society, Interface 10, no. 83 (2013): 20120999, 10.1098/rsif.2012.0999. PMC3645408 23516062

[30] J. Lewis , G. Freisinger , X. Pan , R. Siston , L. Schmitt , and A. Chaudhari , “Changes in Lower Extremity Peak Angles, Moments and Muscle Activations During Stair Climbing at Different Speeds,” Journal of Electromyography and Kinesiology 25, no. 6 (2015): 982–989, 10.1016/j.jelekin.2015.07.011. PMC4658237 26443891

[31] E. Martinez‐Valdes , F. Negro , D. Falla , A. M. De Nunzio , and D. Farina , “Surface Electromyographic Amplitude Does Not Identify Differences in Neural Drive to Synergistic Muscles,” Journal of Applied Physiology 124, no. 4 (2018): 1071–1079, 10.1152/japplphysiol.01115.2017. 29420155

[32] S. Uzlasir , B. Tayfur , E. Işıkdemir , and A. Tayfur , “Comparison of Muscle Activation and Proprioception During Landing at Different Angles Between Individuals With Chronic Ankle Instability and Healthy Controls,” BMC Musculoskeletal Disorders 26, no. 1 (2025): 929, 10.1186/s12891-025-09212-8. PMC12505775 41063064

[33] D. M. Halliday , J. R. Rosenberg , A. M. Amjad , P. Breeze , B. A. Conway , and S. F. Farmer , “A Framework for the Analysis of Mixed Time Series/point Process Data—Theory and Application to the Study of Physiological Tremor, Single Motor Unit Discharges and Electromyograms,” Progress in Biophysics and Molecular biology 64, no. 2–3 (1995): 237–278, 10.1016/S0079-6107(96)00009-0. 8987386

[34] C. M. Laine and F. J. Valero‐Cuevas , “Intermuscular Coherence Reflects Functional Coordination,” Journal of Neurophysiology 118, no. 3 (2017): 1775–1783, 10.1152/jn.00204.2017. PMC5596118 28659460

[35] T. W. Boonstra , A. Danna‐Dos‐Santos , H. B. Xie , M. Roerdink , J. F. Stins , and M. Breakspear , “Muscle Networks: Connectivity Analysis of EMG Activity During Postural Control,” Scientific Reports 5, no. 1 (2015): 17830, 10.1038/srep17830. PMC4669476 26634293

[36] M. D. Smith , B. Vicenzino , R. Bahr , et al., “Return to Sport Decisions After an Acute Lateral Ankle Sprain Injury: Introducing the PAASS Framework—An International Multidisciplinary Consensus,” British Journal of Sports Medicine 55, no. 22 (2021): 1270–1276, 10.1136/bjsports-2021-104087. 34158354

